# Near-infrared spectroscopy and multivariate analysis as effective, fast, and cost-effective methods to discriminate *Candida auris* from *Candida haemulonii*

**DOI:** 10.3389/fchem.2024.1412288

**Published:** 2024-07-10

**Authors:** Ayrton L. F. Nascimento, Anthony G. J. de Medeiros, Ana C. O. Neves, Ana B. N. de Macedo, Luana Rossato, Daniel Assis Santos, André L. S. dos Santos, Kássio M. G. Lima, Rafael W. Bastos

**Affiliations:** ^1^ Laboratório de Química Biológica e Quimiometria, Instituto de Química, Universidade Federal do Rio Grande do Norte, Natal, Brazil; ^2^ Laboratório de Uso Comum, Centro de Biociências, Universidade Federal do Rio Grande do Norte, Natal, Brazil; ^3^ Laboratório de Pesquisa em Ciências da Saúde, Universidade Federal da Grande Dourados, Dourados, Brazil; ^4^ Laboratório de Micologia, Instituto de Ciências Biológicas, Universidade Federal de Minas Gerais, Belo Horizonte, Brazil; ^5^ National Institute of Science and Technology in Human Pathogenic Fungi, Ribeirão Preto, Brazil; ^6^ Instituto de Microbiologia Paulo de Góes, Universidade Federal do Rio de Janeiro, Rio de Janeiro-RJ, Brazil

**Keywords:** *Candida auris*, *Candida haemulonii*, near-infrared spectroscopy, principal component analysis-linear discriminant analysis, successive projections algorithm-linear discriminant analysis, genetic algorithm-linear discriminant analysis, multivariate analysis

## Abstract

*Candida auris* and *Candida haemulonii* are two emerging opportunistic pathogens that have caused an increase in clinical cases in the recent years worldwide. The differentiation of some *Candida* species is highly laborious, difficult, costly, and time-consuming depending on the similarity between the species. Thus, this study aimed to develop a new, faster, and less expensive methodology for differentiating between *C*. *auris* and *C. haemulonii* based on near-infrared (NIR) spectroscopy and multivariate analysis. *C. auris* CBS10913 and *C. haemulonii* CH02 were separated in 15 plates per species, and three isolated colonies of each plate were selected for Fourier transform near-infrared (FT-NIR) analysis, totaling 90 spectra. Subsequently, principal component analysis (PCA) and variable selection algorithms, including the successive projections algorithm (SPA) and genetic algorithm (GA) coupled with linear discriminant analysis (LDA), were employed to discern distinctive patterns among the samples. The use of PCA, SPA, and GA algorithms associated with LDA achieved 100% sensitivity and specificity for the discriminations. The SPA-LDA and GA-LDA algorithms were essential in selecting the variables (infrared wavelengths) of most importance for the models, which could be attributed to binding of cell wall structures such as polysaccharides, peptides, proteins, or molecules resulting from yeasts’ metabolism. These results show the high potential of combined FT-NIR and multivariate analysis techniques for the classification of *Candida*-like fungi, which can contribute to faster and more effective diagnosis and treatment of patients affected by these microorganisms.

## Introduction

*Candida* is a heterogeneous genus of yeasts, which may or may not undergo dimorphic transition to pseudohyphae or hyphae (invasive forms). The main pathogenic species of *Candida* in humans are *C. albicans, C. parapsilosis, C. glabrata, C. krusei, and C. tropicalis*, and additionally *C. haemulonii* infections have been reported more frequently since the year 2000, especially in tropical areas (Bastos et al., 2021; Françoise et al., 2023). These fungi can be part of the human body’s microbiota, and are either commensal or mutualistic. However, due to changes in the host (such as pregnancy, aging, prematurity, chronic diseases, and stress) or extrinsic factors (use of antibiotics, corticosteroids, contraceptives, antiblastic drugs, surgical interventions, trauma, and burns), it can transition to a parasitic stage, causing infectious diseases collectively called candidiasis (Colombo et al., 2013). In addition to these classically known species of *Candida*, *Candida auris*, an emerging pathogenic fungus, has become the target of more recent studies due to its ability to infect hospitalized patients, causes outbreak, to manifest severe forms of the disease, to be persistent in the hospital environment and patient’s skin, and to also be resistant to antifungals (Ahmad and Alfouzan, 2021).

*Candida auris* was first described in 2009, when it was isolated from the ear canal of a patient in Japan, hence the name “*auris*.” Since then, *C. auris* infections have been reported in more than a dozen countries, including the United States, Canada, Colombia, Germany, India, Israel, Japan, Kenya, Norway, Pakistan, Spain, South Africa, South Korea, United Kingdom, Venezuela, Kuwait, Oman, and, recently, in Brazil (Carvajal et al., 2021; de Almeida et al., 2021). *C. auris* can be recovered from various clinical specimens, including sterile body fluids, ear, wounds, and mucocutaneous swabs. However, the main clinical manifestations are invasive and bloodstream infections (Rudramurthy et al., 2017).

Correctly identifying *C. auris* presents a challenge due to its close phylogenetic proximity to other species, such as those in the *Candida haemulonii* complex. Consequently, *C. auris* and the *C. haemulonii* complex share several phenotypic characteristics, complicating their differentiation (Osei Sekyere, 2018). Both species typically display yeast-like growth on standard laboratory media, forming smooth, creamy colonies with similar morphological features, as observed under microscopy. Furthermore, they exhibit resistance to multiple classes of antifungal drugs, including azoles, echinocandins, and polyenes, which complicates treatment strategies and emphasizes the importance of precise identification (Osei Sekyere, 2018).

Distinguishing between *C. auris* and *C. haemulonii* poses significant challenges in clinical laboratories. Conventional phenotypic assays, such as biochemical profiling and morphological characterization, often fail to provide conclusive results due to overlapping traits and variations within the *Candida* genus. Moreover, the absence of species-specific diagnostic markers complicates accurate differentiation, leading to misidentification and potential treatment failures. In fact, misidentifications by biochemical methods are frequent, even with updated databases (Gómez-Gaviria et al., 2023). Therefore, a more accessible, simple, and effective identification becomes essential for studying these multidrug-resistant microorganisms to recognize their specific characteristics. Precisely, there is a need for a more accurate diagnosis for treating infections more quickly and efficiently, without prescribing incorrect medications that ultimately may generate drug-resistant microorganisms. Thus, efforts are needed for discoveries and development of new methods for identification and diagnosis of microorganisms in order to fill gaps and offer medical professionals more possibilities, agility, or precision, depending on their needs.

Infrared spectroscopy is a vibrational technique that has the ability to analyze biological systems, as complex molecules such as proteins, lipids, carbohydrates, and nucleic acids exhibit distinct vibrational behaviors according to their structural and molecular conformation (Neves et al., 2016). Through the emission of electromagnetic radiation in the near-infrared (NIR)—a smaller portion of the infrared spectrum between 900 and 2600 nm—a rapid and accurate diagnosis can be obtained for pathogens isolated from hospital environments and patients (de Sousa Marques et al., 2013).

The use of NIR in recent years has proven to be highly effective for the analysis of various organic, inorganic, and biological substances. NIR offers several advantages, such as rapid identification of various parameters. This efficiency enhances the accuracy of diagnoses, preventing erroneous sample identifications (Cebrián et al., 2021). As NIR is a non-destructive technique requiring little or no sample preparation, it also reduces environmental damage by avoiding or minimizing the use of reagents, which often cause harm to nature. However, for biological samples, this technique itself may not provide sufficient specificity in the search for biomarkers, as many biomolecules are contributing to the entire signal, leading to a large amount of complex data. On the other hand, multivariate analysis has proven to be effective in overcoming this disadvantage (Neves et al., 2016).

There are two classes of multivariate analysis techniques for pattern recognition: unsupervised and supervised methods. The former aims to detect similarities and differences within a dataset composed, for example, of spectra from different classes without prior information about the class to which they belong. Principal component analysis (PCA) is the most popular unsupervised method. On the other hand, in supervised methods, there is prior information of different classes. They are based on two successive steps: first, samples whose class is known are used to build a model with suitable parameters that optimize the discrimination between data from different classes, and then, unknown samples are assigned to an appropriate class using the parameters optimized during the first stage. Linear discriminant analysis (LDA) is an effective supervised approach (Lasalvia et al., 2022).

All measured spectra can be represented as a dataset or matrix X, with n rows corresponding to measured samples and m columns, each corresponding to the spectral signal for a specific wavenumber value. The first objective of PCA is to reduce the dimensionality of large datasets by finding new variables, which are linear functions of those in the original dataset, which successively maximize variance and are uncorrelated with each other (Lasalvia et al., 2022).

LDA is based on a linear transformation of m variables describing n samples belonging to different classes so that samples of the same class are close, but samples from different classes are distant from each other. This goal is achieved through a mathematical classification algorithm (based on calculating the Mahalanobis distance between samples for each class) that maximizes the distance between the means of the classes, while minimizing the variance within each class. Thus, a predicted class is assigned to each sample. After building the classification model, it is used to allocate new and unknown samples to the most likely class. However, the LDA method is generally restricted to problems with few dimensions and cannot be applied when the number of spectral variables is greater than the number of samples (m < n) due to the risk of overfitting since the large number of variables have a high collinearity/redundancy (Morais et al., 2019). This problem can be solved by combining LDA with algorithms that reduce these dimensions, such as PCA, successive projection algorithm (SPA), and genetic algorithm (GA) (José et al., 2005; Lasalvia et al., 2022).

The objectives of this work are to overcome the difficulties of differentiation between two much related species of *Candida*, *C. auris* and C*. haemulonii,* with a new reliable, fast, and relatively less costly method for optimizing diagnoses and methods of research for identifying these yeasts.

## Methodology

### Microorganisms and growth

We used two strains of *Candida auris* and *Candida haemulonii*: *C. auris* CBS10913 from the Westerdijk Institute collection and *C. haemulonii* CH02, provided by Dr. André Dos Santos. *C. haemulonii* CH02 was isolated from a patient and identified phenotypically using CHROMagar *Candida* (CHROMagar Company) and VITEK 2 (bioMérieux) with the YST card. Additionally, it was identified by sequencing the ITS1-5.8S-ITS2 gene (Ramos et al., 2015).

The yeast strains were cryopreserved in brain heart infusion (BHI) growth medium supplemented with 10% glycerol at −80°C until required for experimentation. For experimental procedures, fungi from frozen stocks underwent two successive subcultures on Sabouraud dextrose agar (SDA) for 48 h at 37°C, followed by another cultivation cycle on SDA under the same conditions. Subsequently, isolated colonies were subjected to NIR spectroscopy. The experiment was conducted across 15 plates, with three colonies selected from each plate to generate spectra. In total, 45 colonies per strain were analyzed.

### Preparation of samples for NIR spectra acquisition

One of the advantages and suggestions of this study was the acquisition of spectra without any sample preparation. Using a transflectance probe, each spectrum was obtained by placing the probe directly above the plates containing the colonies. The samples could be used for further analysis following the above method by other researchers, avoiding time loss and reagent consumption.

### Obtaining near-infrared spectra

The 90 colonies, 45 per species, were subjected to NIR spectroscopy by a Fourier transform spectrometer ARCspectro ANIR (ARCoptix, Switzerland) with a 99% reflectance reference underneath, in the region between 900 and 2,600 nm. The detector gain was adjusted to extreme, at one scan, and a Boxcar filter was applied every 10 nm in triplicate (isolated colonies) to obtain as much variability within the same sample and among different samples.

### Multivariate analysis of infrared data

MATLAB software (MathWorks Inc, Natick, MA, USA) was used to import the dataset, perform pretreatment, and construct multivariate classification models (PCA-LDA, SPA-LDA, and GA-LDA). A total of 30 samples were separated for model training and 15 for testing, applying the Kennard–Stone algorithm for infrared spectra (Kennard and Stone, 1969), i.e., a proportion of 70%–30% for training and testing, respectively. Training samples were used to build and optimize the models (selection of variables using the SPA and GA algorithms), while the test samples were used to evaluate their classification using LDA.

A dataset with many variables can be problematic for LDA classification since the probability functions between classes can spread and overlap very easily. Therefore, the number of variables can be simplified by performing data reduction. PCA is a well-known method for reducing the number of variables, creating new ones called principal components, which are linear combinations of the original variables, in which the spectral matrix X is decomposed as follows:

$$X=TP^{t}+E,$$

where X is the I × J data matrix, T is the I × A matrix of score vectors (representing the sample projection in the new space), the score vectors are orthogonal ( $T^{t}T=diag\left(\lambda_{a}\right)$ and $\lambda_{a}$ are the eigenvalues of the matrix $X^{t}X$ ), P is the J × A matrix of loading vectors (weights of the variables), and E is the *I* × J residual matrix. *I* is the number of objects, J is the number of variables, and A is the number of calculated components (de Sousa Marques et al., 2013).

One strategy to avoid overfitting in the SPA-LDA and GA-LDA models is to use a validation set to guide variable selection. The optimal number of variables for SPA-LDA and GA-LDA was determined from the minimum of the cost function G calculated for a given validation dataset as (de Sousa Marques et al., 2013)

$$G=\frac{1}{N_{v}}\sum_{n=1}^{N_{v}}g_{n},$$

where $N_{v}$ is the number of validation samples and $g_{n}$ is defined as

$$g_{n}=\frac{r^{2}\left(x_{n},m_{I\left(n\right)}\right)}{\min_{I\left(m\right)\neq I\left(n\right)}r^{2}\left(x_{n},m_{I\left(n\right)}\right)},$$

where I(n) is the true class index for the *n*th validation object $x_{n}$ ; the numerator $r^{2}\left(x_{n},m_{I\left(n\right)}\right)$ is the squared Mahalanobis distance between the object $x_{n}$ (of class index I(n)) and the sample mean $m_{I\left(n\right)}$ of this true class; and the denominator is the squared Mahalanobis distance between object $x_{n}$ and the error center of the nearest class, i.e., the wrong class (Neves et al., 2016).

To obtain a discriminant profile, the LDA classification score $\left(L_{ij}\right)$ is calculated for a given class k by the following equation:

$$L_{ik}={\left(x_{i}-{\bar{x}}_{k}\right)}^{T}\Sigma_{pooled}^{-1}\left(x_{i}-{\bar{x}}_{k}\right)-2\log_{e}\pi_{k,}$$

where $x_{i}$ is an unknown measurement vector for the sample i; ${\bar{x}}_{k}$ is the measurement vector for the mean of the classes k; $\Sigma_{pooled}$ is the pooled matrix of covariance; and $\pi_{k}$ is the prior probability of class k (Neves et al., 2016).

The GA algorithm was set to a minimum of 40 generations and a maximum of 80 generations. Crossover and mutation probabilities were set to 60% and 10%, respectively, and repeated three times, starting from different random initial populations.

Accuracy (number of samples correctly classified considering true and false negatives) (Morais et al., 2020), sensitivity (SENS, the confidence that a positive result for a sample of the label class is obtained) (Morais et al., 2020), specificity (SPEC, the confidence that a negative result for a sample of the non-label class is obtained) (Morais et al., 2020), G-score (model performance not accounting for class size) (Morais et al., 2020), and AUC (area under the curve that measures the relation between true positives and false positives, giving the probability of a model to classify a random positive example higher than a random negative example) (López et al., 2013) were calculated as important quality parameters in test evaluation.

$$Accuracy\;\left(\%\right)=\frac{TP+TN}{TP+FP+TN+FN}\times 100,$$

$$SENS\left(\%\right)=\left(\frac{TP}{TP+FN}\right)\times 100,$$

$$SPEC\left(\%\right)=\left(\frac{TN}{TN+FP}\right)\times 100,$$

$$Gscore=\sqrt{SENS\times SPEC,}$$

$$AUC=\frac{1+TPR-FPR,}{2}$$

where

$$TPR=\left(\frac{TP}{TP+FN}\right),$$

and

$$FPR=\left(\frac{FP}{FP+TN}\right).$$

TPR and FPR are true-positive rate (percentage of positive instances correctly classified) and false-positive rate (percentage of negative instances misclassified), respectively, FN is defined as false negative, and FP is false positive. TP and TN are defined as true positive and true negative, respectively (López et al., 2013).

**FIGURE 1:**
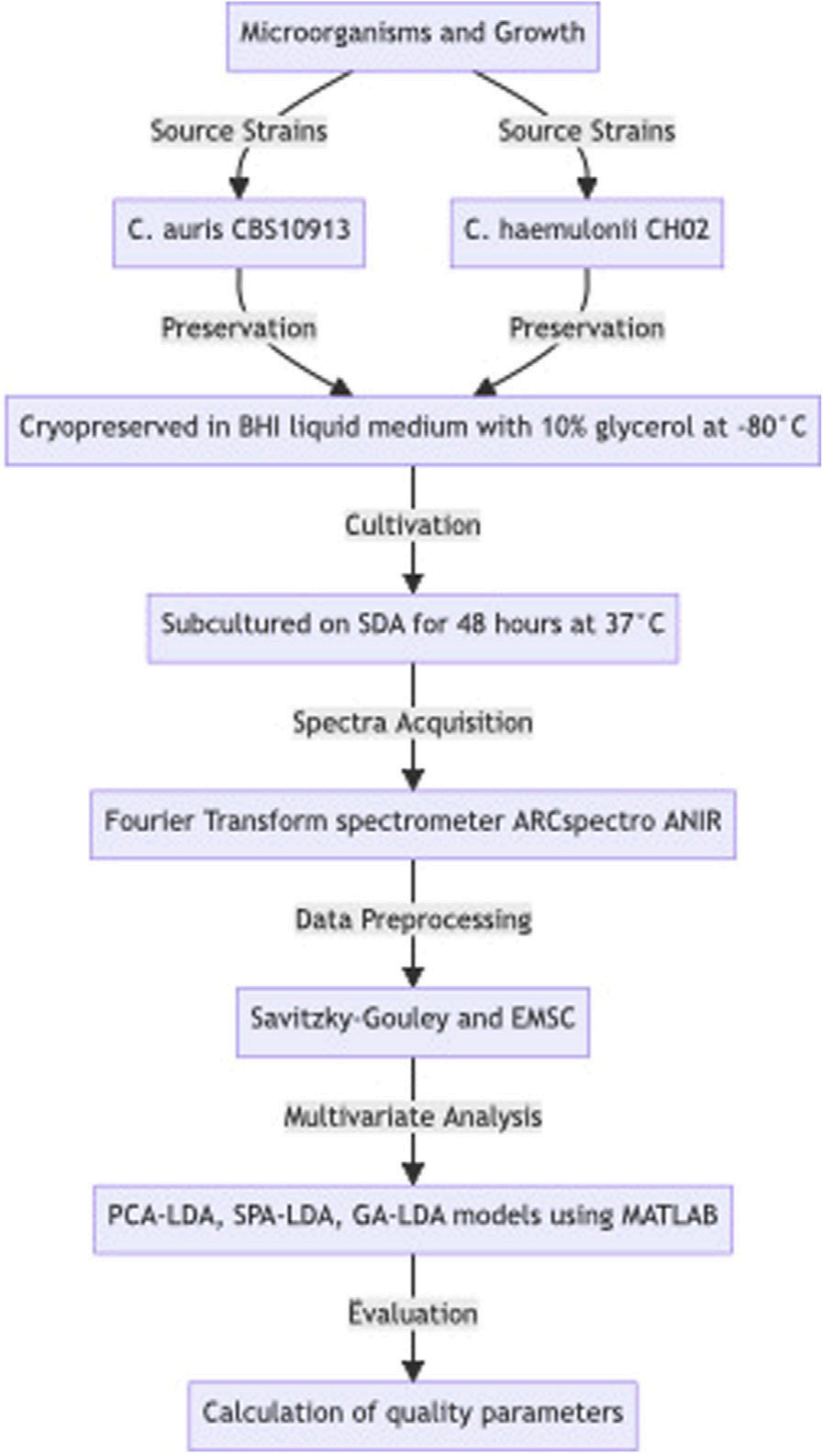
Flowchart describing the methodology.

Herein, for all calculations, *C. auris* CBS1093 samples were considered the positive class (“disease group”) and *C. hemulonii* CH02 samples as the negative class (“control group”). Figure 1 shows a flowchart describing the methodology of this work.

## Results and discussion

**FIGURE 2:**
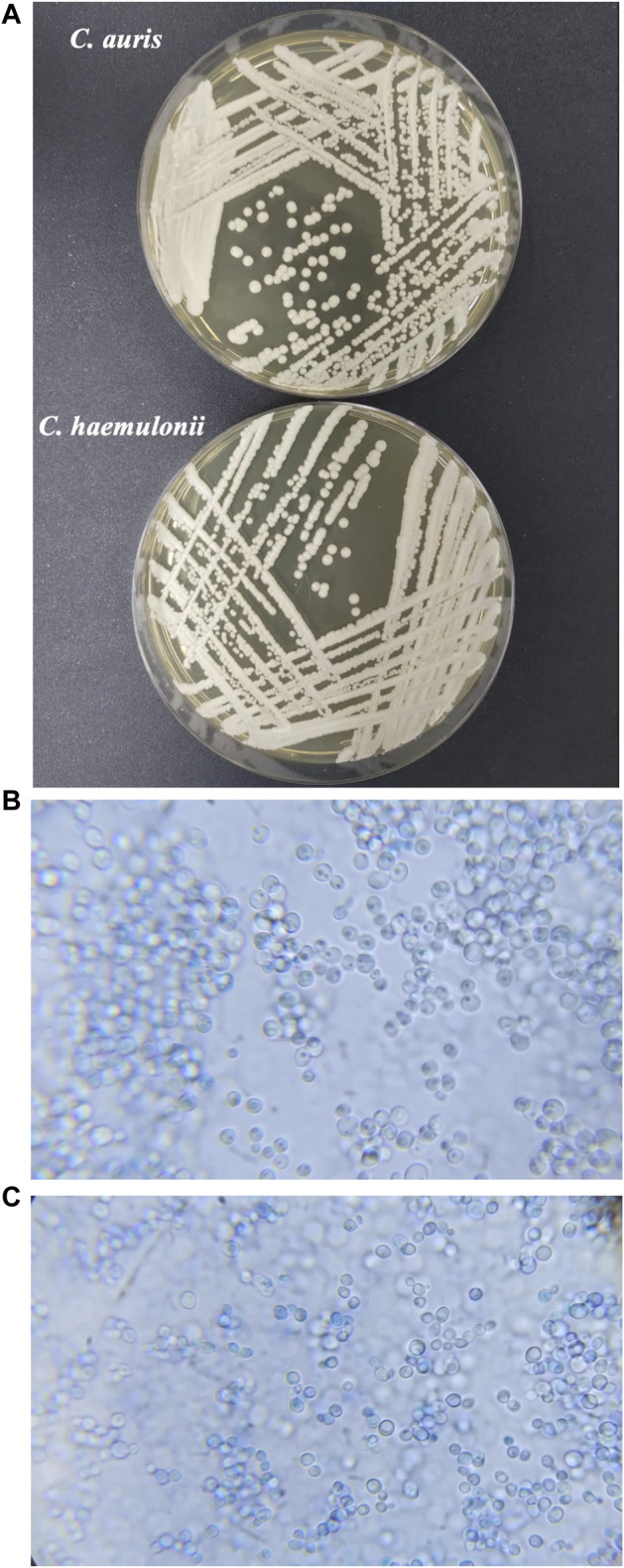
**(A)** *Candida auris* CBS10913 and *C. haemulonii* CH02 samples for visual comparation. Microscopy of *C. auris* CBS10913 **(B)** and **(C)** *C. haemulonii* CH02 in methylene blue at ×100 magnification by an optical microscope.

Differentiating between *C. auris* and *C. haemulonii* complex species is challenging in clinical practice because these species have phylogenetic proximity and share similar morphological and physiological characteristics. Figure 2 shows the macro-morphology of the colonies (Figure 2A) and the micro-morphology of the cells (Figures 2B, C) of the two species, highlighting how they are morphologically similar. Both species typically display yeast-like growth on standard laboratory media, forming smooth, creamy colonies with similar morphological features observed under microscopy (Figure 2).

**FIGURE 3:**
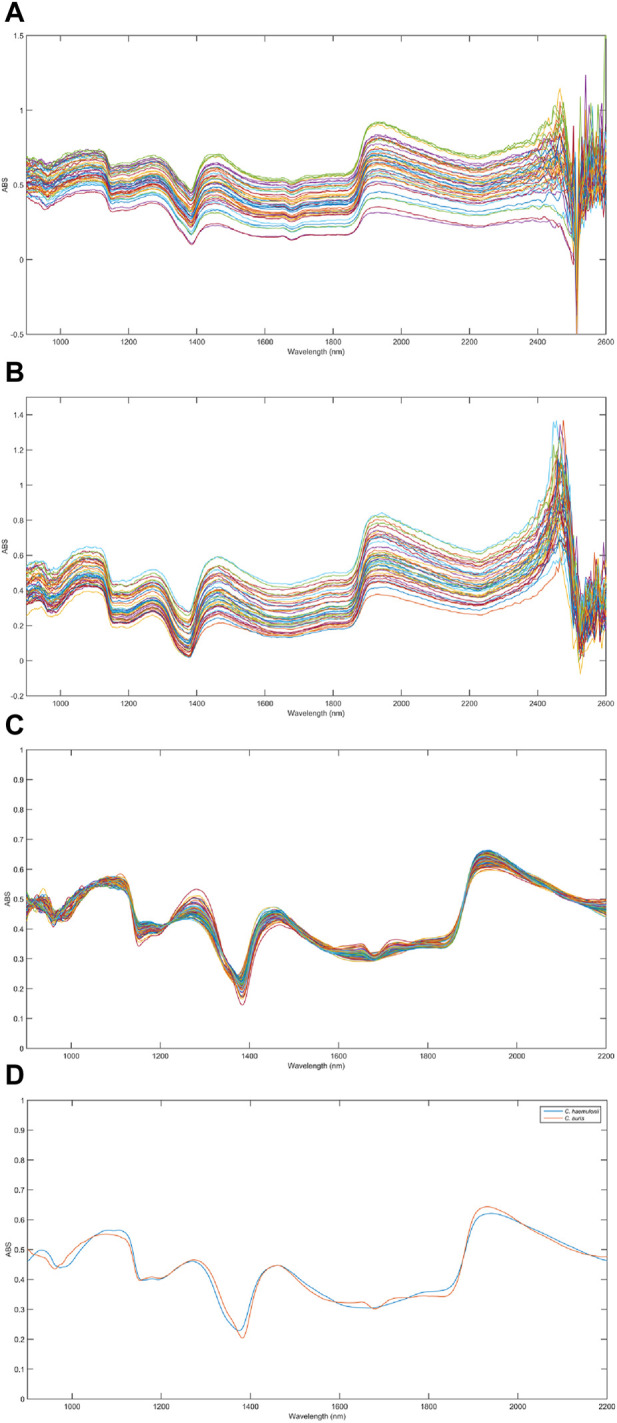
Raw infrared spectra for **(A)** *Candida auris* CBS10913 and **(B)** *Candida haemulonii* CH02 samples. **(C)** All pretreated spectra for both species and **(D)** mean pretreated spectra for both species were *C. auris* CBS10913 and *C. haemulonii* CH02 are represented by red and blue lines, respectively.

Figures 3A, B show the raw NIR spectra obtained for individual colonies (Figure 2A) of *C. auris* CBS10913 and *C. haemulonii* CH02, respectively. Figure 3C shows all spectra, for both species, after pretreatment. Figure 3D presents the mean pretreated spectra for both species. The region between 2,200 and 2,600 nm from the raw NIR spectra (Figures 3A, B) showed a poor signal-to-noise ratio (S/N) and was removed before building the discrimination models as it may not provide any useful information. As pretreatments, Savitzky–Golay smoothing filter (5 points window) and extended multiplicative scatter correction (EMSC) were applied, both from the PLS ToolBox (Eigenvector Research, Inc., Manson, WA, USA) in the MATLAB environment, to improve the signal and correct it for light scatterings, respectively. Figure 3D shows the spectral similarity between classes. The spectra are slightly shifted downward, relative to each other, but these are mean spectra, and distinguishing an isolated spectrum from another to separate the sample class is very difficult, necessitating computational analysis to identify markers responsible for differences between species.

**FIGURE 4:**
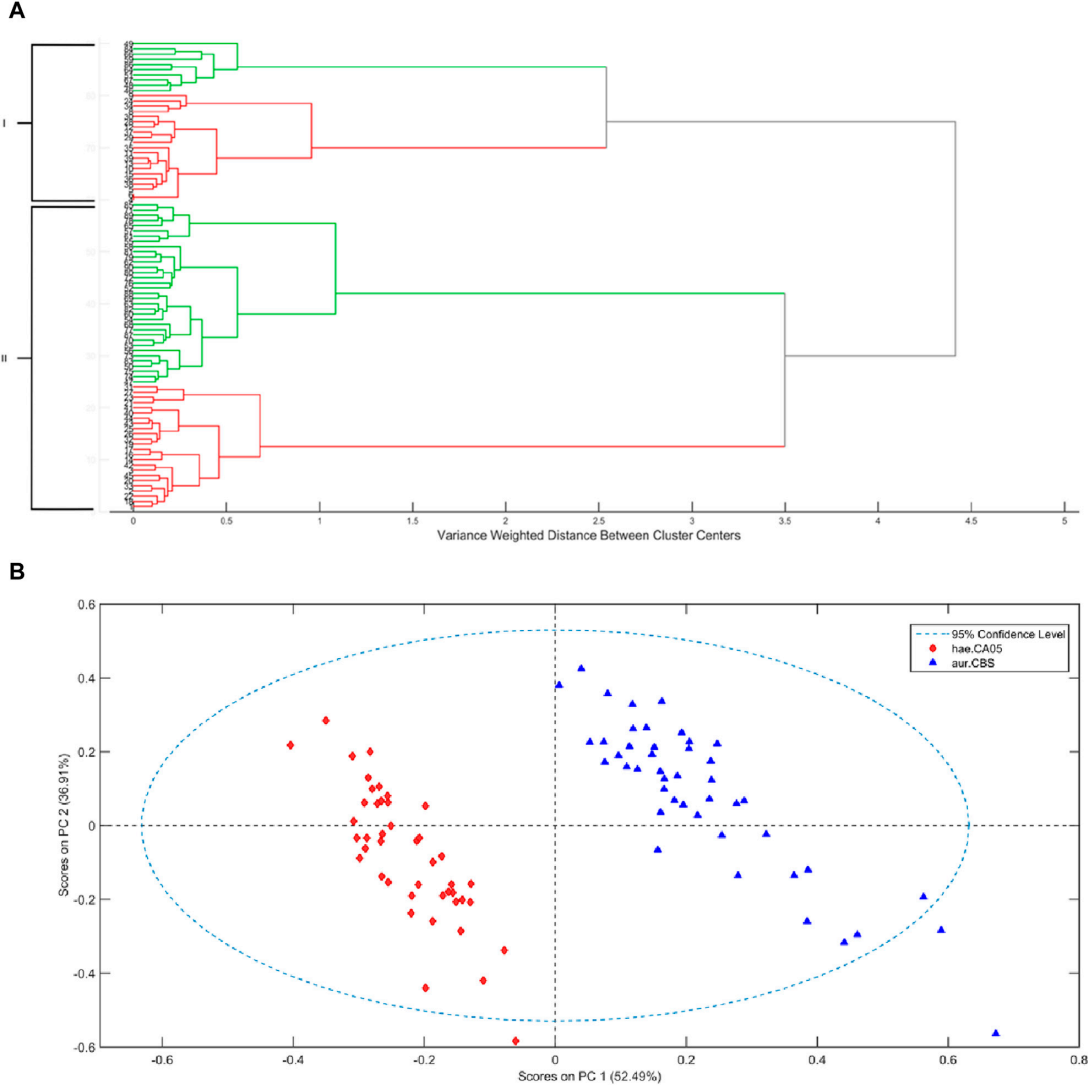
HCA **(A)** of NIR for *Candida auris* CBS10913 (red) and *C. haemulonii* CH02 (green). PCA scores for *Candida auris* CBS10913 and *C. haemulonii* CH02 **(B)**.

First, an exploratory analysis was carried out by applying hierarchical cluster analysis (HCA) and PCA to observe the behavior of samples regarding their division in clusters related to the species of *Candida* without the need of any prior class information. To perform the HCA, it is necessary to have a metric function for sample distances (in this case the Mahalanobis distance was applied), a linkage criterion among groups (Ward’s linkage was used), and the agglomerative hierarchical clustering technique was used. Initially, each sample is considered an individual cluster, and subsequently, pairs of clusters are merged based on their similarities. This process results in a dendrogram, a two-dimensional tree-like diagram that shows the groups of merged samples (Neves et al., 2021). The dendrogram in Figure 4A shows the presence of two main clusters, where the two classes of *Candida* are mixed, highlighting their similarities. When the NIR data are analyzed by PCA, two distinct groups for each class are formed, as shown in the PCA score plots (Figure 4B). Although the first two principal components of PCA accounted for 89.4% of the explained variance and were able to separate the two distinct groups for each class, a mathematical function is still needed to predict the classes so that the model can be used for unknown samples in future diagnoses.

### *Candida* auris CBS10913 vs. *Candida* haemulonii CH02

**FIGURE 5:**
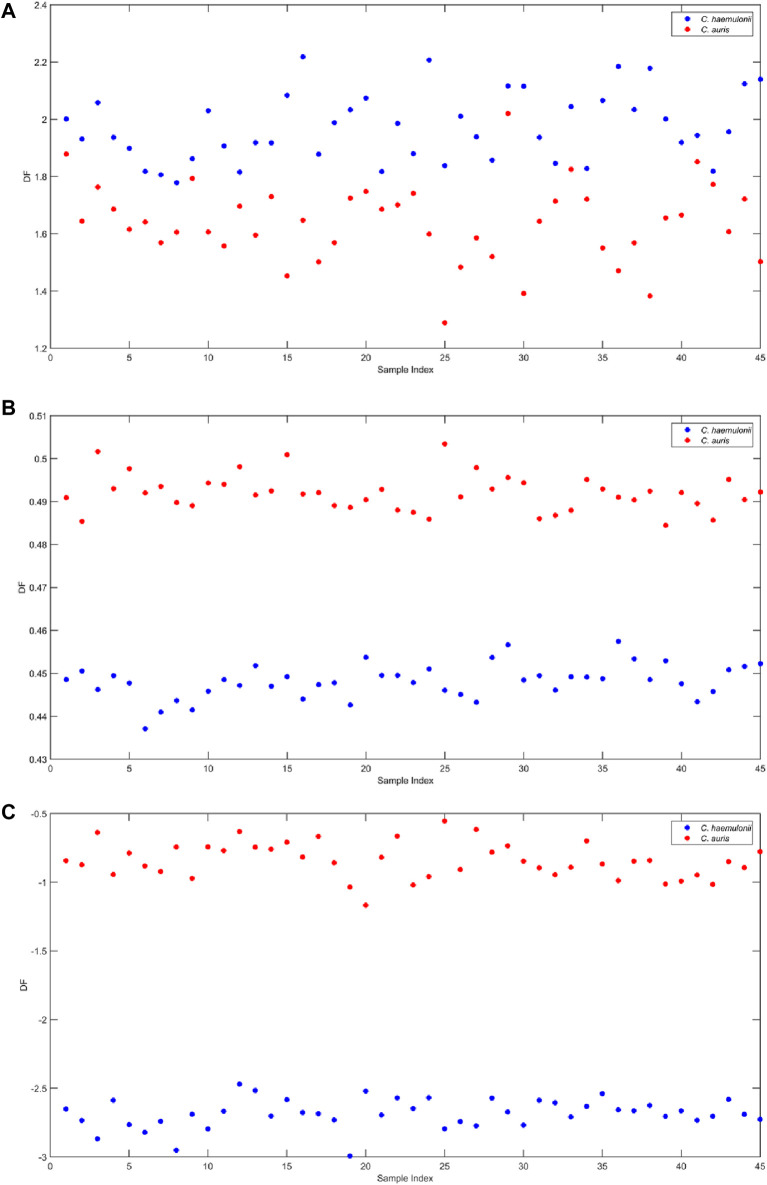
**(A)** PCA-LDA discriminant function over the NIR spectra for *C. auris* CBS10913 and *C. haemulonii* CH02. **(B)** SPA-LDA discriminant function over the NIR spectra for *C. auris* CBS10913 and *C. haemulonii* CH02. **(C)** GA-LDA discriminant function over the NIR spectra for *C. auris* CBS10913 and *C. haemulonii* CH02.

Figure 5A represents the Fisher discriminant function for PCA-LDA. Only two PCs were needed to explain 92.22% of data variation and showed good separation. Although the PCA-LDA model satisfactorily discriminated the classes, considering attempts to correlate classification results with biomarker searches by attributing functional groups and/or chemical bonds reflected in NIR wavelengths, PCA-LDA may not be the best option available due to its nature of creating a new space and losing the original information. An alternative to that issue is using SPA and GA algorithms to select the most important variables (wavelengths) for the model. Figures 5B, C show the discriminant function over the spectral observation points for SPA-LDA and GA-LDA, respectively. In addition to selecting the ideal number of variables, these algorithms were able to increase the visual separation.

**FIGURE 6:**
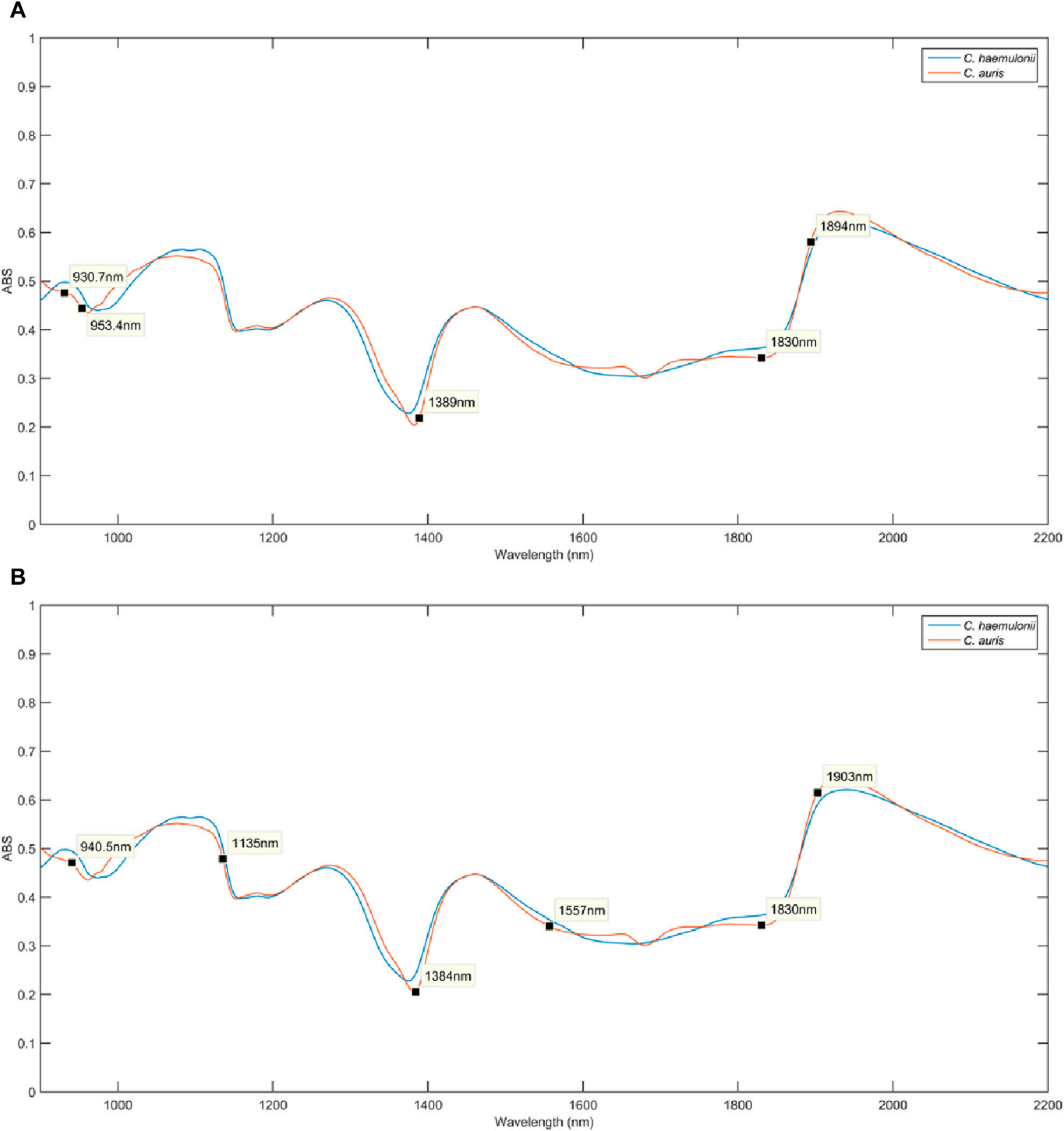
**(A)** Variable selection of SPA-LDA over the mean pretreated spectra for *Candida auris* CBS10913 (orange line) and *C. haemulonii* CH02 (blue line). **(B)** Variable selection of GA-LDA over the mean pretreated spectra for *Candida auris* CBS10913 (orange line) and *C. haemulonii* CH02 (blue line).

Table 1 displays the confusion matrix showing real and predicted classes and the number of samples in which each algorithm classified them. For this case, the positive class is represented by *C. auris* CBS10913 and the negative class is represented by *C. haemulonii* CH02. As shown in Table 2, the three methods were capable of classifying the species with maximum sensitivity (meaning their capability to correctly identify patients positive for *C. auris* infection) and specificity (meaning the capability of models to identify patients positive for *C. hemulonii* infection). Although PCA-LDA results are equally satisfactory, SPA-LDA and GA-LDA have identified the most important variables for the models, as depicted in Figures 6A, B.

**TABLE 1:** Table of confusion from PCA-LDA, SPA-LDA, and GA-LDA models.

Actual class		*C. auris*	*C. haemulonii*
PCA-LDA			
	*C. auris*	15	0
	*C. haemulonii*	0	15
SPA-LDA			
	*C. auris*	15	0
	*C. haemulonii*	0	15
GA-LDA			
	*C. auris*	15	0
	*C. haemulonii*	0	15

**TABLE 2:** Quality performance values from PCA-LDA, SPA-LDA, and GA-LDA models.

Quality performance feature	PCA-LDA	SPA-LDA	GA-LDA
Accuracy (%)	100	100	100
Sensitivity (%)	100	100	100
Specificity (%)	100	100	100
G-score	100	100	100
AUC	1	1	1

SPA-LDA selected the following wavelengths in nanometers: 931, 953, 1389, 1830, and 1894. These wavelengths are associated with the third overtone of C-H bonds in hydrocarbons, alcohol secondary overtones, C-H combination bands associated with long aliphatic molecules, O-H/C-H combination bands of polysaccharides, and C=O stretch second overtone of carboxylic acids, respectively. GA-LDA selected 940, 1135, 1384, 1557, 1830, and 1903. These wavelengths are associated with the second overtone of alcohol combination bands, hydrogen bond secondary amide second overtone, C-H combination bands associated with long aliphatic molecules, hydrogen bond secondary amide first overtone, O-H/C-H combination bands of polysaccharides, and hydrogen bond in P-OH group first overtone, respectively (Siesler et al., 2001; Workman and Weyer, 2008). The wavelengths selected by SPA-LDA and GA-LDA correspond highly to the subtle spectral differences observed between the species, which could be attributed to important intrinsic variations such as different polysaccharides, peptides, or protein structures in the cell wall and metabolic products (Garcia-Rubio et al., 2019; Oliver et al., 2020).

The discriminatory ability of the models presented herein is not only equivalent but also outstanding when considering the results achieved for all the quality performance parameters evaluated in this study, as seen in Table 2. However, it is important to note that, among the other reduction algorithms in this study, SPA has the advantage of being deterministic, i.e., it always returns the same selected variables each run. In contrast, GA may vary slightly in the selection of variables across different runs due to its random nature, while the original variables are not present in PCA anymore, as it generates two new latent variables to explain the variance within the data. This aspect of SPA might play an important role when considering the assignment of the main variables responsible for the discrimination and their correlation with groups of molecules that may act as biomarkers. The area under the curve (AUC) of prediction samples for SPA-LDA, which evidences the model’s capacity to truly classify and not just give random results, accounted for the maximum value of 1.

In addition to differentiating two emergent yeast species, our results are very important in the context of clinical practice. Individuals who are at risk of acquiring *C. auris* infections are primarily hospitalized and nursing home patients. These patients generally have comorbidities that, together with the multidrug-resistant nature of the yeast, contribute to the lethality of this infection, which can reach up to 60% of cases (Wang et al., 2018). Additionally, *Candida haemulonii* has emerged as an opportunistic pathogenic fungus associated with nail infections, onychomycosis, paronychia, vaginal candidiasis, blood infections, and several fungemia related to catheters, osteitis, and outbreaks in ICUs (Leite-Jr et al., 2023). The rapid identification of these species, facilitated by the method employed in this study, enables the prompt application of targeted treatments.

Standard methods to diagnose candidiasis, in general, can be laborious or highly costly. For example, through direct mycological examination (slides of biological material, whether oral, vaginal, or bloodstream mucosa) treated with KOH solution and stained with an appropriate dye and/or by cultivation in specific mycological media, with subsequent identification of the pathogen by microscopic examination of its structures, through automation, MALDI-TOF, or molecular methods (Colombo et al., 2013; Montes et al., 2019). On the other hand, our study provides a more efficient, simple, and cost-effective method to discriminate between *Candida auris* and *Candida haemulonii* since there is no need for highly specialized personnel, sample preparation, or very expensive materials and equipment.

Previous studies have recognized the potential of NIR spectroscopy in mycology. For instance, Cebrián et al. (2021) presented data demonstrating the utility of NIR in identifying substances produced by molds, while Santos et al. (2010) demonstrated the capability of identification and characterization of filamentous fungi and yeast forms through Fourier transform infrared spectroscopy (Santos et al., 2010; Cebrián et al., 2021). Essendoubi et al. (2005) used Fourier transform infrared spectroscopy with hierarchical clustering analysis to distinguish *Candia* species (*Candida albicans*, *Candida glabrata*, *Candida parapsilosis*, *Candida tropicalis*, *Candida krusei*, and *Candida kefyr*) (Essendoubi et al., 2005). However, our study marks the first instance of employing NIR spectroscopy in conjunction with supervised algorithms for this specific purpose. This approach has been shown to be more precise than unsupervised methods, as discrimination is based on a mathematical function that calculates the probability of a sample belonging to a given class, i.e., creates a model. Notably, our methodology successfully differentiated between two *Candida* species, highlighting its efficacy.

## Conclusion

This study successfully differentiated between the closely related species *C. auris* CBS10913 and *C. haemulonii* CH02 using NIR spectroscopy combined with multivariate analysis. The SPA-LDA and GA-LDA models achieved 100% accuracy, sensitivity, and specificity in distinguishing these species. These models identified crucial spectral features, demonstrating robust discriminatory capabilities. SPA-LDA showed a significant advantage in biomarker identification due to its deterministic nature. The selected wavelengths correlated with subtle spectral variations, potentially linked to polysaccharides, peptide or protein structures, and metabolic products. These findings suggest that NIR spectroscopy, coupled with advanced multivariate analysis, can offer a rapid, accurate, and cost-effective method for yeast identification, improving clinical diagnostics and treatment strategies. Further validation with larger datasets is recommended to extend this approach to other *Candida* species.

## Data Availability

The raw data supporting the conclusion of this article will be made available by the authors, without undue reservation.

## Abbreviations

NIR, near-infrared ; FT-NIR, Fourier transform near-infrared ; PCA, principal component analysis; SPA, successive projection algorithm; GA, genetic algorithm; LDA, linear discriminant analysis; S/N, signal-to-noise ratio; HCA, hierarchical cluster analysis; SDA, Sabouraud dextrose agar; BHI, brain heart infusion; EMSC, extended multiplicative scatter correction; SENS, sensitivity; SPEC, specificity; G-Score, geometric score; AUC, area under the curve; TP, true positive; TN, true negative; FP, false positive; FN, false negative; TPR, true-positive rate; FPR, false-positive rate; PCs, principal components.
